# Scholarly publishing literacy and perceptions of predatory publishing among educational partners at a chiropractic college: a case report

**DOI:** 10.5195/jmla.2026.2226

**Published:** 2026-10-01

**Authors:** Jennifer Smith, Edward W. Murphy, Stacie A. Salsbury

**Affiliations:** 1 jennifer.smith@das.iowa.gov, Southeast District Consultant, State Library of Iowa, Davenport, IA and former Head Librarian of Public Services, David D. Palmer Health Sciences Library, Palmer College of Chiropractic, Davenport, IA; 2 edward.murphy@palmer.edu, Branch Manager Librarian, David D. Palmer Health Sciences Library, Palmer College of Chiropractic Florida Campus, Port Orange, FL; 3 stacie.salsbury@palmer.edu, Professor, Palmer Center for Chiropractic Research, Palmer College of Chiropractic, Davenport, IA

**Keywords:** Predatory Journals as Topic, Scholarly Communication, Information Literacy, Chiropractic, Education, Professional, Libraries, Medical, Library Surveys

## Abstract

**Background:**

Chiropractors must find, appraise, and apply high quality, peer-reviewed information sources to make evidence-based clinical practice decisions. This case report describes perceptions of scholarly publishing practices, with a focus on predatory and deceptive publishing, among chiropractic educational partners at one chiropractic college.

**Case Presentation:**

An anonymous, online, cross-sectional survey was administered to a convenience sample of 2,476 faculty, staff, and students. The 35-item questionnaire elicited publishing experience, critical appraisal skills, knowledge of scholarly and predatory publishing, and perceptions of chiropractic journals and information resources. Respondents (n=204, 111 students, 36 faculty, 16 administrators, and 41 staff) provided usable survey responses, though skipped items were common. Twenty-six percent had published one or more peer-reviewed publications. Most self-rated themselves as novice or intermediate in their ability to perform literature searches (87%) or critically appraise research articles (82%). Forty-six percent to 69% had not read or were unsure of the quality of scientific evidence for 11 chiropractic-related journals. Fifty-seven percent were unsure or unfamiliar with predatory journals and 51% lacked confidence in recognizing these journals. Predatory practices identified included insufficient or fast peer review, editorial board and impact factor issues, publication fees, plagiarized articles, and unsolicited request for paper submissions. Sixty-one percent requested training or resources on identifying quality academic journals for publication or research.

**Conclusion:**

Chiropractic educational partners were unfamiliar with most chiropractic journals and had little familiarity with predatory publishing. Targeted training on identifying quality academic publications, conducting literature reviews and critically appraising research articles is recommended.

## BACKGROUND

Chiropractors need the ability to locate, evaluate, and use high quality, peer-reviewed information sources to make evidence-based clinical practice (EBCP) decisions [1–3]. Chiropractors must have sufficient information literacy skills, including knowledge of scholarly publishing practices and core chiropractic journals and reference materials [4,5]. The profession has struggled against antiscience sentiments from its founding and more recently saw unproven claims raised purporting protective mechanisms of chiropractic treatment during the COVID-19 pandemic [6,7]. While original research and scholarship generated by chiropractic faculty and students is an area of growing emphasis [8–12], chiropractic educational partners may lack research expertise and scholarly writing skills [13–15].

Chiropractic educational partners have engaged in numerous initiatives to increase practitioner and student knowledge and skills with EBCP, noting favorable gains in attitudes and use [2]. However, persistent barriers remain, including perceived lack of time, insufficient clinical evidence, and issues with locating, critically appraising, or interpreting the literature [2,4,16–23]. These barriers may lead chiropractors and students to rely on lower quality information sources, like websites and trade publications, instead of peer-reviewed sources of scientific evidence [2].

Faculty across higher education institutions often face intense pressures to publish [24,25] which may lead some faculty to publish their works within predatory journals [26,27]. Grudniewicz et al. within *Nature* offered a comprehensive, consensus definition of predatory journals, describing them as “characterized by false or misleading information, deviation from best editorial and publication practices, a lack of transparency, and/or use of aggressive and indiscriminate solicitation practices” [28]. Predatory publishing is a persistent problem that promotes poor scientific practices and lowers editorial standards across academic fields [29].

In this case report, the authors describe a project designed to understand the training and support needs of potential health science library users at one U.S.-based chiropractic college regarding scholarly publishing, with an emphasis on journal quality and predatory publishing. Our team sought to answer the following questions:

1. What experiences have chiropractic educational partners in our institution had with scholarly publishing, including predatory publishing?
2. What resources do faculty, students and staff in our institution use and consider as reputable sources of information about chiropractic?

## CASE PRESENTATION

This single-institution, cross-sectional survey evaluated perceptions of scholarly publishing literacy among educational partners at Palmer College of Chiropractic campuses in Iowa, Florida and California. The Doctor of Chiropractic program enrolls on a trimester basis, with hundreds of new student users introduced to the David D. Palmer Health Sciences Library each term. The library has two locations and is home to the largest collection of chiropractic works in the world, which includes books, journals, audiovisual materials, models, tools, and databases to support EBCP.

This project was timely and needed for several reasons. First, library resources were undergoing re-organization related to shifts in campuses and academic programs. Secondly, scholarly publishing was encouraged on multiple levels. Chiropractic students could participate in research honors programs and were encouraged to co-author research papers and case reports in collaboration with faculty. Academic and clinical faculty and staff members could receive financial incentives for authorship of scholarly publications but reported difficulties with selecting appropriate journals for their manuscripts. Thus, we hoped to identify knowledge gaps regarding predatory publishing and chiropractic journal quality.

We submitted the protocol to the Palmer College of Chiropractic Institutional Review Board, which deemed the project exempt. The 35-item online survey was adapted from prior research on publication literacy in academic health sciences [30,31] and designed within SurveyMonkey (San Mateo, CA, USA) using a feature that prevented recipients from completing the survey more than once. Most items were multiple choice, with some open-ended questions provided. The survey asked respondents to rank relevant journals based on perceived quality of scientific evidence. Journal titles were selected based on history within the profession [5]; availability in the library collection; topical focus on chiropractic or spine care; impact factor (IF) as an indicator of rank within the field [32]; and known sources for faculty publications. Each question was optional, and survey completion was voluntary. Six librarians pre-tested the survey to ensure its clarity and flow (Appendix A). No incentives were offered. Descriptive statistics were summarized, including the number and percentages of responses. Skipped items ranged from 0 to 78 respondents for the quantitative questions, with more items skipped in the final third of the survey, suggesting survey fatigue [33]. Open-ended questions were coded by 2 authors (JS, SAS) using a conventional approach to content analysis [34].

We emailed survey invitations to a convenience sample of 2,476 potential library users from July to August 2024. In total, 210 people responded to the invitation, consent was received for 208 surveys, with 204 containing usable data (8.2% response rate). Respondents included 111 chiropractic students, 36 faculty (including 1 resident), 41 staff members, and 16 administrators.

### What Experiences Have Chiropractic Educational Partners Had with Scholarly Publishing, Including Predatory Publishing?

Chiropractic educational partners in our institution described themselves as novice (24%), intermediate (32%), advanced (33%), or expert (10%) in their knowledge of chiropractic. Respondents rated their ability to perform literature reviews as novice (44%), intermediate (43%), advanced (10%), and expert (3%). Novice (36%), intermediate (46%), advanced (14%), and expert (4%) ratings were reported for critically appraising research articles. While 74% reported no publishing experience, 53 respondents had published at least 1 peer-reviewed journal article, including 7 staff, 9 administrators, 11 students, and 26 faculty.

When asked if they were familiar with predatory journals, 43% of respondents said yes, 42% said no, and 15% were unsure; among those replying no/unsure, 76% were students, 54% staff, 22% faculty, and 13% administrators. Respondents were divided evenly between confidence (49%) or not confident (51%) about identifying a predatory journal, with non-confidence reported by 64% of students, 50% staff, 35% faculty, and 13% administrators. 21% of respondents had received an email invitation to submit papers, 12% had colleagues/friends recommend articles, 7% were invited to join editorial boards, and 6% had read articles. The majority had not used Think, Check, Submit (93%) to assess if a journal title was predatory [35].

Table 1 depicts the characteristics of predatory journals identified in a survey checklist and described in open-ended responses. Respondents identified many characteristics ascribed to deceptive publications, such as no/insufficient peer-review (71%), no/fake editorial board (68%), promise of fast publication (59%), high publication fees (55%), no/use of fake impact factors (53%), publication of plagiarized articles (53%), and unsolicited requests for articles (52%). Open-ended responses (n=120) added the characteristics of low quality of journal content, misinformation or ulterior motives for publishing decisions, and unethical practices.

**Table 1:** Characteristics of predatory journals identified by chiropractic educational partners

Predatory journal characteristic	% Respondents		Open-ended response example
	Survey Items	Open-Ended	
No/insufficient peer-review	70%	25%	Journal that charges you but does not perform proper evaluation of the submitted work
No/fake editorial board	68%	0	--
Promise of fast publication	59%	3%	A business disguised as a journal that charges high publication costs in return for fast publication times due to a lack of proper peer-review.
High fees/publication on payment	55%	34%	A journal enticing authors to publish on their journals for a fee to receive peer review status or accelerate the publication of an article.
No/fake impact factors	53%	0	--
Plagiarized articles	53%	0	--
Unprofessional layout	53%	0	--
Unsolicited request for articles	52%	12%	Bush league journals that seek out authors to include in their publications (and charge fees).
Articles with poorly written English	48%	0	--
Low content quality	0	21%	Journal prioritizes collection of fees from authors at the expense of conducting appropriate peer review and/or ensuring the scientific integrity and/or quality of published articles (if they are published at all).
Misinformation or ulterior motive	0	18%	Journal that goes out of their way to spread misinformation
Unethical	0	6%	Journal that looks legit, but has practices that draw in articles, and then say you have to pay to get it published or does not properly cite the articles in it.
Publication schedule^#^	1%	0	--
Reputable publisher^#^	1%	0	--
Indexed in MEDLINE^#^	0.7%	0	--
Not sure/do not know	16%	17%	I have never heard the term predatory journal.
Skipped item	n=64	n=10	

# Item included as example of standard academic publishing practice

### What resources do faculty, students and staff in our institution use and consider as reputable sources of information about chiropractic?

Chiropractic academic journals were the most popular information sources for research or professional development (71%). DynaMed/medical platforms (54%) and health sciences journals (53%) also were referenced.

Within these publications and platforms, systematic reviews/meta-analyses (63%), case reports/series (56%), clinical practice guidelines (51%), clinical trials (51%), and review articles (50%) were the most frequently consulted sources. When evaluating resources for quality, respondents’ top criteria were reported as the journal itself (64%), study design (57%), and publication status (i.e., peer-reviewed vs pre-print) (51%). Most (86%) correctly defined open-access as a manuscript published in a journal with peer-review and which is freely distributed online. Only 54% selected the correct definition of an article pre-print as a manuscript published in a digital version, but which has not gone through the peer-review process.

News about chiropractic, that is, information about the profession that is not necessarily based on research, was sourced from scientific journals and research databases (66%), social media (39%), professional or governmental websites (37%), DynaMed/medical platforms (32%), televised reports (10%), and Research Square/pre-print platforms (2%), while ‘other’ sources included professional associations, conferences, institutional communications, chiropractic educational institution websites, holistic health magazines, blogs, or podcasts. Nearly half (48%) reported checking 2 to 5 references before sharing information with colleagues, while 19% checked at least 1 additional resource.

Figure 1 depicts chiropractic journals rated on the perceived quality of scientific evidence. Journals rated as higher quality by greater than 30% of respondents were *Journal of Manipulative and Physiological Therapeutics* (IF 1.5), *Spine* (IF 4.6), *Journal of Chiropractic Education* (IF 0.7), and *Chiropractic and Manual Therapies* (IF 2), which generally aligned with the journal impact factors. Journals ranked of average or lower quality were often indexed only in the *Index of Chiropractic Literature* [36] and did not have IF ratings. These findings indicated respondents’ unfamiliarity with many chiropractic journals (46% to 69% having no-to-little knowledge of various journals), with 61% interested in receiving training on how to identify quality academic journals.

**Figure 1:**
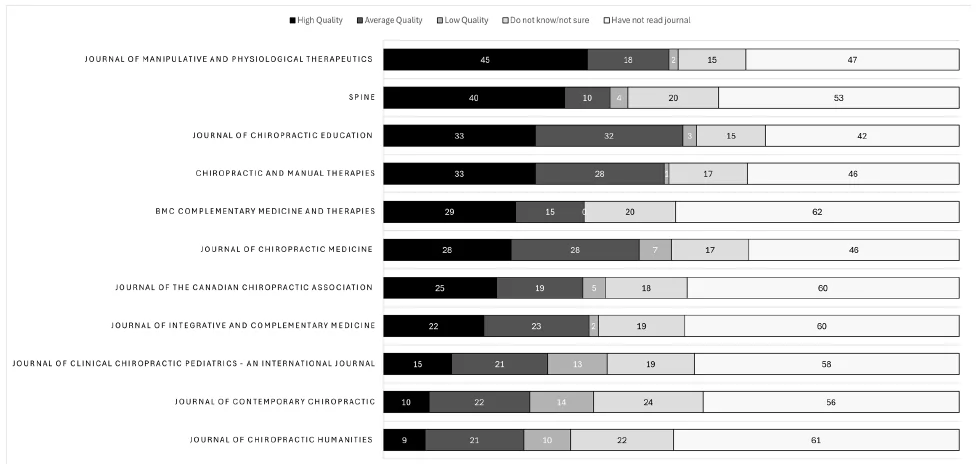
Chiropractic journal titles rated on the quality of their scientific evidence

## DISCUSSION

This case report explored perceptions of scholarly publishing literacy among educational partners at one chiropractic college. Only 26% of respondents reported authorship of a peer-reviewed publication, although this included students, staff, faculty and administrators. Most respondents, especially students, considered themselves at the novice or intermediate level of ability in conducting literature searches and critically reviewing journal articles, which is similar to other studies within chiropractic [2,13,17]. Informal and formal training initiatives, support, and mentorship in scholarly activities may be helpful to develop these skill sets, as was noted in a recent scoping review of faculty scholarship in chiropractic [8]. Chiropractic educational partners were somewhat unexperienced with deceptive publishing practices, as only 43% were familiar with predatory journals and 49% expressed confidence in recognizing such publications. Swanberg and colleagues reported similar knowledge gaps as 40% of university and medical faculty in their institution could not correctly identify predatory journals [37]. Faculty across academic settings report high concerns and low familiarity with predatory publishers [38,39]. Although lacking confidence, respondents correctly identified common characteristics of predatory journals [40]. Librarians can instruct educational partners about the characteristics of predatory publishers, including the strategies used by such journals to solicit articles and the problems when such articles are integrated into clinical practice guidelines and similar reviews [37,41,42]. Librarians also can ensure that library collections do not contain these journals [43].

According to the 2018 Council on Chiropractic Education (CCE) Accreditation Standards meta-competency 6 “Information and Technology Literacy”, doctor of chiropractic programs have the curricular objective to prepare students to “locate, critically appraise and use relevant scientific literature and other evidence” with the outcome that “students will be able to use relevant scientific literature and other evidence to inform patient care” [44]. Chiropractic students have reported deficits in their research literacy, such as in their ability to access peer-reviewed journals, identify study designs, and select successful search strategies [13,45]. Respondents displayed low level of familiarity with many chiropractic journals (46% to 69%), as was noted in other recent publications [27,46]. Further, only 2 titles received quality rankings from the majority of faculty and administrators. While respondents reported using a variety of information sources, many had a high reliance on case reports, non-peer-reviewed resources, and social media. Not surprisingly, 61% of respondents expressed interest in training and resources on information literacy and scholarly publishing.

Health sciences librarians working in chiropractic academic institutions may be well placed to lead such efforts, as chiropractic students are receptive to educational interventions offered within the library setting [13,45,47]. Librarians in our institution have implemented initiatives aimed at increasing student and faculty awareness of high-quality publications, including regular newsletters highlighting recently published chiropractic research in prominent health sciences journals. Workshops and resources on scholarly publishing, including predatory publishing and how to select information resources may be warranted. Although not specifically surveyed in this study, we also foresee the need to incorporate information on artificial intelligence (AI) literacy into these tools and trainings [48].

One strength of this case report was its focus on scholarly publication literacy, including knowledge of predatory publishing practices, among chiropractic educational partners, a population for which limited information is available. This survey had its limitations. Our response rate was 8.4%, which is consistent with low responses reported in similar surveys in health sciences settings [37,49]. Incentives, shorter surveys, survey administration timing, or tailored project information to subgroups might improve response rates. Targeted deployment, such as types of staff invited, may have improved the response. Selection bias is present in any convenience sample, so our results might reflect those with an interest in scholarly publication. Our team modified published survey instruments [30,31]. This modification could have benefited from pre-testing with students to ensure comprehension of item wording and to decrease the overall number of items, which might have led to fewer skipped questions. Future studies might build upon this single-institution case report to explore scholarly publication literacy at chiropractic training programs worldwide, which could increase generalizability.

## Data Availability

Data associated with this article are available in the Open Science Framework at https://osf.io/sm8nw/. **Registration:** Open Science Framework, https://osf.io/dspra.

## SUPPLEMENTAL FILES

**Appendix A:** Survey Instrument
