# Supplementary material for: Scholarly publishing literacy and perceptions of predatory publishing among educational partners at a chiropractic college: a case report
Source: J Med Libr Assoc. 2026 Oct 14;114(4):393–400. doi: 10.5195/jmla.2026.2226 (PMC13670217; doi:10.5195/jmla.2026.2226)
Supplement: Supplementary file 1 [file jmla-114-4-393-s01.docx]

**Appendix A. Survey instrument - Scholarly publishing literacy and perceptions of predatory publishing among educational partners at a chiropractic college (Smith, Murphy & Salsbury)**

1. ***Do you agree to participate in this survey?***

- I AGREE to take part in this survey.
- I DECLINE to take part in this survey.

2. **Are you 18 years of age or older?**

- Yes
- No **(No will exclude the participant from entering into the survey)**

3. ***Your home campus is***

- Main
- Florida
- West

4. ***Current Profession?***  ***Mark only one oval.***

- Chiropractic student
- Resident or other training position
- Faculty member
- Administrator
- Other staff member

5. ***Students: How much of your chiropractic education have you completed so far?***
   - Less than year 1
   - Between year 1-2
   - Between year 2-3
   - In my final year
   - Not a student
6. ***How do you define your gender?***

- Male
- Female
- Transgender
- Another gender identity
- I prefer not to say

7. ***What is your age in years?***  ________
8. ***Is English your primary language?***

- Yes
- No

***9. Publishing experience. How many peer-reviewed articles have you published? Mark only one oval.***

- 0
- 1
- 2-5
- 6-10
- >10

***10. Have you heard of predatory journals?***  ***Mark only one oval.***

- Yes
- No
- Do not know/not sure.

***11. How do you define a predatory journal?***

***12. Have you used Beall’s List to see if a journal is a “predatory” journal? Mark only one oval.***

- Yes
- No
- Do not know/not sure.

***13. Have you ever used the Think, check, submit website? Mark only one oval.***

- Yes
- No
- Do not know/not sure

A predatory journal is “is an exploitative academic publishing business model that involves charging publication fees to authors without checking articles for quality and legitimacy and without providing editorial and publishing services that legitimate academic journals provide, whether open-access or not. They are regarded as predatory because scholars are tricked into publishing with them, although some authors may be aware that the journal is of inadequate quality or even fraudulent.”  (Kabulo, Kanmounye, Ntshindj, et al. 2022.)

***14. How have you interacted with a predatory journal? Mark all that apply.***

- A colleague/friend suggested an article or the journal.
- I received an email invitation to submit a paper.
- I was contacted by a predatory journal on social media.
- I read articles published in a predatory journal.
- I attended an event organized by a predatory journal.
- I submitted an article for publication.
- I reviewed an article for publication.
- I was asked to join the editorial board.
- I am an editor or editorial board member.
- Do not know/not sure
- Other ____________

***15. How confident are you identifying a predatory journal?  Mark only one oval.***

- Not confident at all
- Slightly confident
- Somewhat confident
- Fairly confident
- Completely confident

***16. Which of the following features are associated with predatory journals?***  ***Mark all that apply.***

- No impact factor or use of fake impact factors
- Indexed in MEDLINE
- Articles with poorly written English
- No or insufficient peer review
- No editorial board or fake editorial board
- Sponsored by a professional organization
- High publication fees or publication upon receipt of payment
- Publication of plagiarized articles
- Unprofessional journal layout
- Established or reputable publisher
- Promise of fast publication or extremely rapid publication process (Within a few days or weeks of submission)
- Unsolicited request for articles
- Consistent publication schedule
- Other _____________________

***17. Have you ever discovered that your article was published in a predatory journal? Mark only one oval.***

- Yes
- No
- Do not know/not sure.
- Not applicable

***18. If you published in a predatory journal, what was your role in the papers published? Mark all that apply.***

- First author
- Second author
- Middle author
- Senior/Last author
- Corresponding author
- Have not published
- Have not published in a predatory journal

***19. If you published in a predatory journal, were you aware it was a predatory journal at***

***the time the article was submitted.*** ***Mark only one oval.***

- Yes
- No
- Do not know/not sure
- Have not published

***20. Will you submit future articles to predatory journals?***  ***Mark only one oval.***

- Yes
- No
- Do not know/not sure/not intentionally

***21. Please explain your answer to question 20.***

***22. Have you reviewed articles for a predatory journal?***  ***Mark only one oval.***

- Yes
- No
- Do not know/not sure.

***23. What kinds of sources do you primarily use for your chiropractic training, skill building, and/or research?***

***Mark all that apply.***

- Chiropractic academic journals.
- Other health science academic journals such as: The Lancet, Nature, Journal of the American Medical Association (JAMA), British Medical Journal (BMJ), or PLOS ONE.
- Medical platforms such as DynaMed, etc.
- Continuous medical education programs, webinars, conferences, or others provided openly by universities or professional organizations.
- Chiropractic continuing education programs, webinars, or others provided by Palmer College of Chiropractic, not including Palmer Homecoming
- Palmer College of Chiropractic Homecoming
- Palmer College of Chiropractic Faculty In-service
- Open access courses from institutions such as the World Health Organization (WHO).
- Academic courses from Palmer College of Chiropractic
- Academic courses from another college or university.
- Peer discussions with your classmates or other faculty.
- Other ______________________________

***24. What is the main platform you use to get news about chiropractic medicine? Mark all that apply.***

- Social media (Twitter/X, Instagram, Facebook, LinkedIn, etc.)
- Official entities web pages or summaries such as the World Health Organization (WHO), Centers for Disease Control (CDC), National Institutes of Health (NIH), etc.
- Scientific community and news anchors opinions on television.
- Professional/non-governmental organization (NGO) summaries and briefs.
- Scientific journals and research paper databases (PubMed, CINAHL, Cochrane Library, Index to Chiropractic Literature (ICL), ScienceDirect, etc.).
- Pre-publication platforms such as Research Square.
- Medical platforms such as DynaMed, etc.
- Other (please specify)

***25. What kinds of academic papers do you use for updated chiropractic information? Mark all that apply.***

- Case reports/case series
- Clinical trials
- Case control studies
- Systematic literature reviews/meta-analyses
- Review articles
- Cohort studies
- Cross-sectional studies
- Clinical practice guidelines
- Other types of studies

***26. What are the main criteria that you consider when reading a research article or paper?  Mark all that apply.***

- Study design.
- Author affiliation
- Endorsement from institutions
- Publishing status (peer review vs. preprint)
- Number of references used
- Journal where it is published
- Results are new or novel
- Results confirm what is known about chiropractic
- Recommendation from a colleague or trusted source
- Link from social media
- Short summary is available
- Prefer to receive research content by video or podcast
- I don’t read research articles on a regular basis
- Other

***27. What is a preprint?  Mark only one oval.***

- A manuscript that has gone through peer review and published in a digital version, not paper print.
- A manuscript that has been published in a digital version but has not gone through peer review yet.
- A protocol proposal that is published in a journal, approved for other authors to be able to contribute to the study’s design.
- A manuscript approved by one of the reviewers and rejected by another published for a short time for the scientific community to provide feedback.

***28. What is an Open Access article?  Mark only one oval.***

- A manuscript published in a journal with peer review and which is freely distributed online.
- A manuscript that is published in a journal without peer review, that charges for its publication.
- A manuscript that is published in digital version but has not gone through peer review.
- A manuscript that has been accepted by the journal’s reviewers but has not been published yet.

***29. Typically, how many sources do you check before sharing any kind of information?  Mark only one oval.***

- None
- Only one (if it is reliable)
- 2 to 5
- More than 5
- I usually don’t share source with other people

***30. Rate the following journals on the quality of their scientific evidence.*** (Survey grid to rate using the following criteria: “Low Quality”, “Average Quality”, “High Quality”, “Do not know/Not Sure”, “Have not read journal”)

_____ BMC Complementary Medicine and Therapies

_____ Chiropractic and Manual Therapies

_____ Journal of Chiropractic Education (JCE)

_____ Journal of Chiropractic Humanities

_____ Journal of Chiropractic Medicine (JCM)

_____ Journal of Clinical Chiropractic Pediatrics - an International Journal

_____ Journal of Contemporary Chiropractic

_____ Journal of Integrative and Complementary Medicine

_____ Journal of Manipulative and Physiological Therapeutics (JMPT)

_____ Journal of the Canadian Chiropractic Association (JCCA)

_____ Spine

***31.Rate your knowledge of performing a literature review. (Mark only one)***

- Novice
- Intermediate
- Advanced
- Expert level

***32. Rate your knowledge of assessing the quality of a research article. (Mark only one)***

- Novice
- Intermediate
- Advanced
- Expert level

***33. Rate your knowledge of chiropractic (Mark only one)***

- Novice
- Intermediate
- Advanced
- Expert level

***34. Would you like training or resources on identifying quality academic journals for publication or research?***

- Yes
- No

***35. Please enter any thoughts you have about using or submitting research publications within the field of chiropractic.***

Some of these questions have been adapted from a survey by Kabulo, Kanmounye, Ntshindj, et al. 2022, others have been adapted from a survey by Nicolalde, Añazco, Jaramillo-Cartwright, et al. 2022.
